# Prediction of stability coefficient of open-pit mine slope based on artificial intelligence deep learning algorithm

**DOI:** 10.1038/s41598-023-38896-y

**Published:** 2023-07-25

**Authors:** Shuai Wang, Zongbao Zhang, Chao Wang

**Affiliations:** 1 School of Civil Engineering, Liaoning Technical University, Fuxin, 123000 Liaoning China; 2 College of Mining, Liaoning Technical University, Fuxin, 123000 Liaoning China

**Keywords:** Solid Earth sciences, Geology, Geomagnetism

## Abstract

The mining of open pit mines is widespread in China, and there are many cases of landslide accidents. Therefore, the problem of slope stability is highlighted. The stability of the slope is a factor that directly affects the mining efficiency and the safety of the entire mining process. According to the statistics, there is a 15 percent chance of finding landslide risk in China’s large-scale mines. And due to the expansion of the mining scale of the enterprise, the problem of slope stability has become increasingly obvious, which has become a major subject in the study of open-pit mine engineering. In order to better predict the slope stability coefficient, this study takes a mine in China as a case to deeply discuss the accuracy of different algorithms in the stability calculation, and then uses a deep learning algorithm to study the stability under rainfall conditions. The change of the coefficient and the change of the stability coefficient before and after the slope treatment are experimentally studied with the displacement of the monitoring point. The result shows that the safety coefficient calculated by the algorithm in this paper is about 7% lower than that of the traditional algorithm. In the slope stability analysis before treatment, the safety factor calculated by the algorithm in this paper is 1.086, and the algorithm in this paper is closer to reality. In the stability analysis of the slope after treatment, the safety factor calculated by the algorithm in this paper is 1.227, and the stability factor meets the requirements of the specification. It also shows that the deep learning algorithm effectively improves the efficiency of the slope stability factor prediction and improves security during project development.

## Introduction

During the excavation of open pit mines, it will result in the formation of highly steep side slopes. This way, the original balance state of the land is destroyed, and the rock mass will also be offset due to the force, which makes the slope especially easy to lose its stability. The slope stability of an open pit mine can directly affect the safety of the mine source, as well as the economic benefits of the mine source. In fact, the higher the slope stability, the better. It should follow the principles of economy and safety and determine a reasonable slope angle. If the stability is too high, it will increase the mining of the mine source, thereby reducing the economic benefits of the mine. If the stability is too low, it will make the hillside unstable, and additional hillside reinforcement will be required, which not only affects the production but also increases the cost. Only by obtaining an economical and safe stability factor can the safety of the entire project be ensured, the output efficiency can be ensured, and costs can be saved. Although the technology of selecting mines is also optimized with the progress of society, it is always a high-risk industry, and the safety issue of the stability coefficient during the excavation process is also a problem that scholars have been discussing. Generally speaking, when the stability of a slope is controlled by the same inclined sliding surface, the influence of slope height on its stability is greater than that of the slope gradient. Therefore, the author tries to study the problem of slope stability coefficient prediction of open pit mines from the perspective of artificial intelligence deep learning algorithm, hoping to get an ideal effect.

With the implementation of the country's policy of developing mines, increasing scholars have paid attention to the safety issues in the development process and have done a lot of research on the stability of open-pit slopes. Among them, Zhu, et al. used the method of numerical simulation to study the influence of hydraulic fractures on the slope stability of open-pit coalbed methane well fracturing^1^. But the algorithm he adopted in this paper is not very suitable for the calculation of mining angle. Before it, Besimbaeva, et al. evaluated the slope stability of the open-pit barite mine located in the eastern Anjing mining province and studied the rock mass strength characteristics using two methods^2^. But the evaluation system applied in this paper is not very comprehensive. Later, Kang, et al. conducted a slope stability evaluation for the gold mine and analyzed its probabilistic evaluation and sensitivity^3^. However, he did not discuss the scale classification method adopted in more detail in the article. Christian introduced the stability evaluation of a step slope in an open-pit mine in Peru and obtained the probability density function of the structural plane parameters^4^. But the experiments he conducted in the paper did not take into account possible factors.

After referring to the research results of other scholars, Dehghan and Khodaei calculated the minimum safety factor of 1.20, which provided a stable optimal design for the limit slope of the open pit mine^5^. But he did not refer to the influence of the safety factor when calculating the horizontal displacement of the model. Du and Ye proposed to combine the interval fuzzy number with its reliability to obtain mixed information and then used the similarity measure for slope stability decision^6^. However, when he defines the logical relationship, the content of the elaboration is not very detailed. Singh, et al. used simulation software to evaluate the overall stability factor of the overburden dump^7^. But he did not include the impact of the weak soil in his conclusion. Natural and artificial soil slope damage is a complex phenomenon that can cause serious harm in many countries around the world. As a result, millions of dollars’ worth of public and private property were destroyed. It is necessary to understand the process leading to slope failure and predict its vulnerability in order to appropriately mitigate the risk of slope failure. Suman attempted to use recently developed artificial intelligence methods such as functional networks (FNs), multiple adaptive regression splines (MARS), and multigene genetic programming (MGGP) to predict the safety factor of slopes^8^. The performance of these AI technologies has been evaluated according to different statistical parameters, such as average absolute error, maximum absolute error, root mean square error, and correlation coefficient, and Nash Sutcliffe efficiency coefficient. Slope stability analysis is one of the most important issues in geotechnical engineering. The development of slope stability analysis closely follows the development of computational geotechnical engineering. Das discussed the application of different recently developed artificial neural network models in slope stability analysis based on the available actual slope failure databases in the literature^9^. He developed different artificial neural network models to classify slopes as stable or unstable (failure) and predict safety factors.

The popularity of artificial intelligence has been very extensive, and deep learning is actually a new research direction of machine learning. It is an algorithm that is closest to the goal of artificial intelligence in machine learning. And it has obtained a lot of results in data mining and other fields, and can help people solve a lot of problems. The innovation of this paper lies in the use of a novel method, deep learning algorithm, to study the slope stability coefficient of open pit mines. In the process of research, a very simple but effective method is used to obtain a large amount of relevant data for analysis. Hope to be able to provide support for future mine excavation work.

## Prediction method of slope stability coefficient of open pit mine

### Side stability of open pit mine

**Figure 1:**
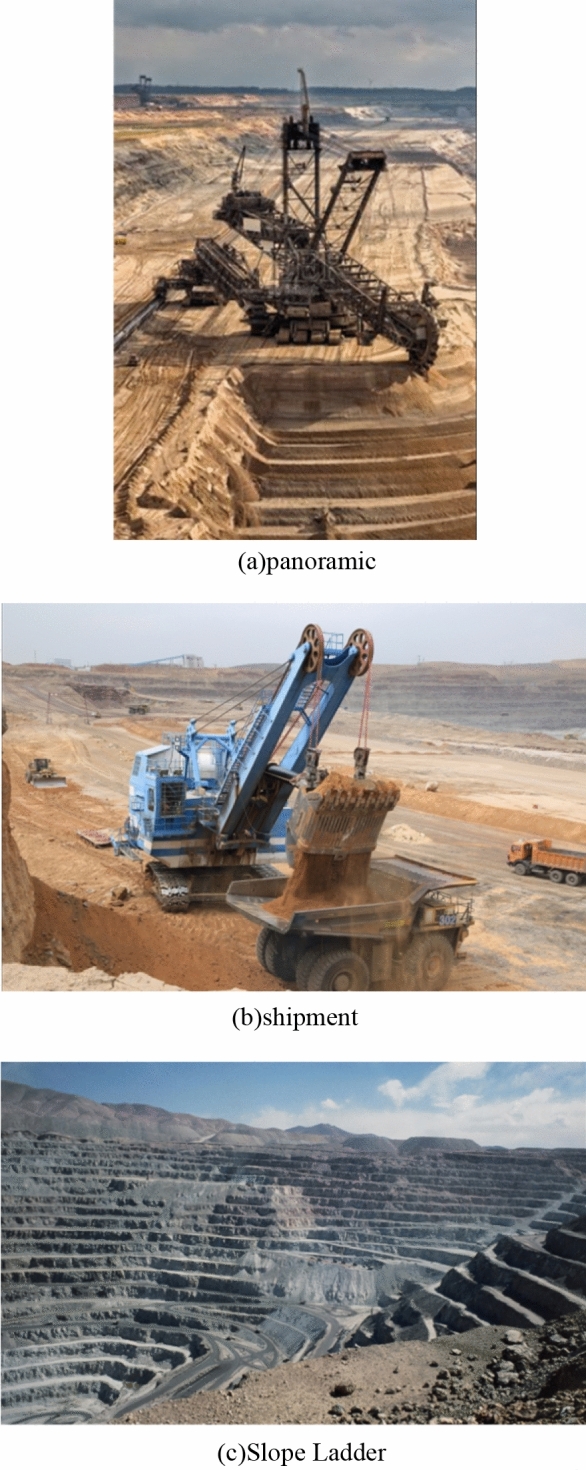
Mining of an open pit mine.

When developing open-pit mines, the limitation of rock slope safety is the main reason that affects mine production efficiency. The proportion of open-pit mining in China is actually very large. The mining of iron ore and fossil raw materials is almost always in the form of open pit mining. During the mining process, the safety of the slope body is the most important. Therefore, in the mining process, it is necessary to increase the final slope angle and ensure the stability of the slope. There will be a very sharp contradiction in the mining process, that is, the larger the final slope angle, the more unstable the slope will be. If this problem is not handled properly, it will seriously affect the safety production of the mine and the economic benefits of the mine. And the mining of open-pit mines is very likely to cause the surrounding environment to become unsafe. Therefore, when mining, it is necessary to ensure safety without reducing the mining speed and reducing the impact on the surrounding environment. Committed to achieving an economical and efficient stripping ratio^10^. The mining of the open pit mine is shown in Fig. 1.

As shown in Fig. 1, in the mining process of the mine, professional tools such as excavators need to be used^11^. And it will form a first-order slope. In fact, open-pit mines in China are still relatively common, and there are many mines in Inner Mongolia and Xinjiang. These mines have very serious stability problems, and many large landslides have occurred. This has caused a lot of economic losses to the mining mines. So the issue of stability has always been an essential issue. At the same time, because the shallow resources are being developed by people, they are constantly decreasing. Therefore, new technologies have been created to develop deep resources. However, this technology will have a very large impact on the entire mine rock formation, the environment will also be damaged, and safety and stability will also be reduced. Although many scholars have improved the stability of mining in different ways, the stability of each mine is an independent quantity, and they have different characteristics. Therefore, it is necessary to determine which method to use for prediction according to the on-site assessment of the mine.Therefore, further research is needed to find better ways to mine mines^12^.

**Figure 2:**
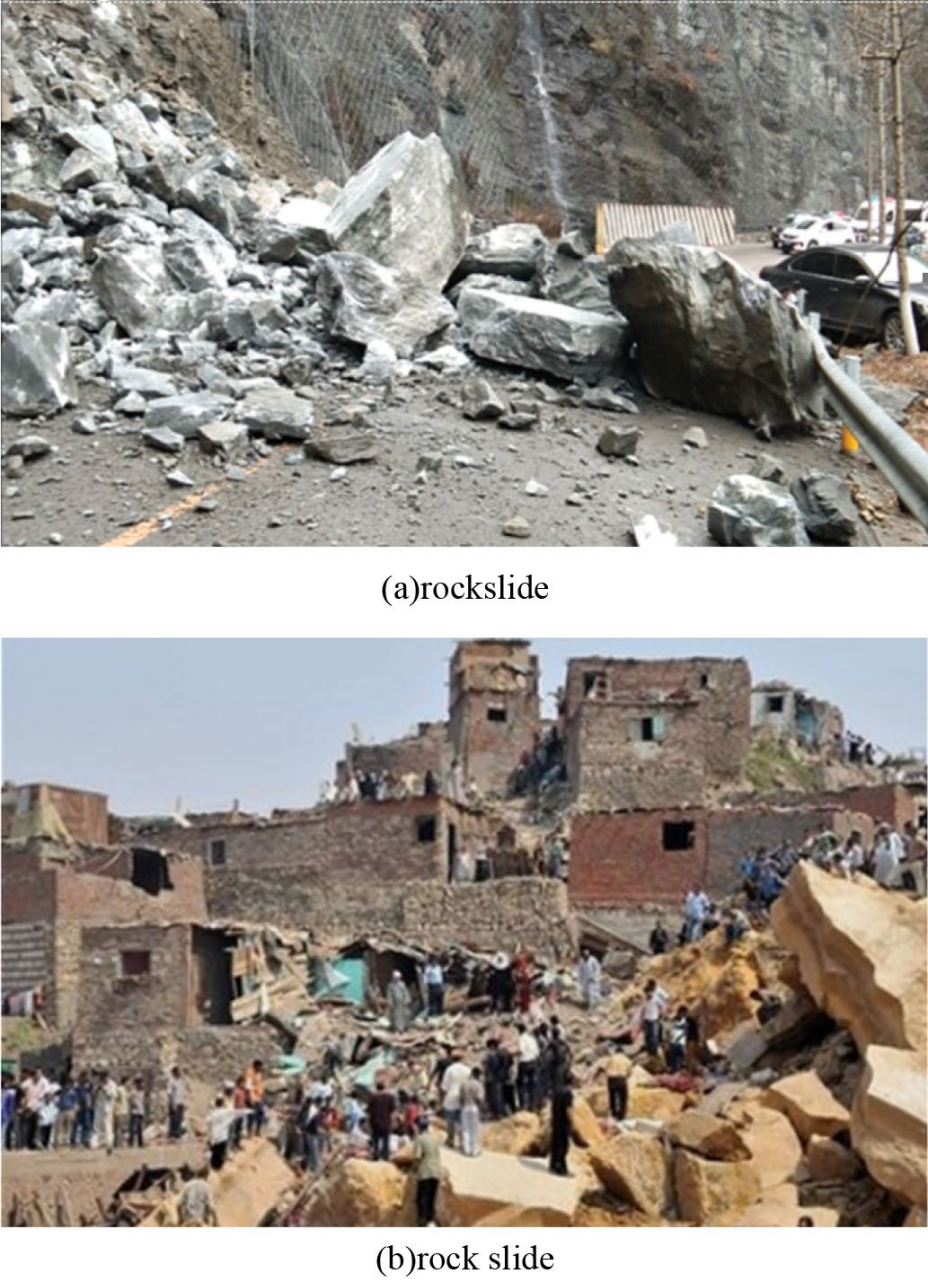
Types of landslide.

If the stability of the slope is not good, it is easy to occur a landslide disaster, which is a very serious problem^13^. It threatens people's property and lives. The occurrence of landslides is generally due to the destruction of rock slopes. Rockslides and rockslides are the main types of rock slope damage as shown in Picture 2:

As shown in Fig. 2, there are two main types of landslides^14^. The first is rock avalanches. It mostly happens on the kind of very steep slopes where the rock breaks apart in chunks and then collapses, tumbling forward. The rock body at the top is often detached and then falls off due to some factors, and accumulates at the foot of the slope. These situations often occur where there are cracks on the top of the slope. Cracks are also created by weathering of rock over time, or by the intrusion of rainwater and prolonged soaking. However, it is also possible that due to changes in temperature, high temperature or shading may cause the rock to loosen. The protective measures taken by general experts are to use artificially reinforced building materials, that is, anchor cables. This way, the impact force of rock mass collapse and sliding can be minimized. The second is rock slip, which is a phenomenon in which the rock mass slides along a certain surface^15^. In fact, the main reason for rock slip is because of too much rainfall. After surface water seeps into the cracks, it will generate hydrostatic pressure, which is the force that promotes the sliding of the soil slope and is detrimental to the stability of the soil slope. Due to the infiltration of rainwater, the rise of river water level, or the impoundment of reservoirs, the groundwater level rises, causing static water pressure to act on the impermeable structural surface of the slope. It acts perpendicular to the structural surface and acts on the slope, weakening the normal stress generated by the weight of the sliding mass on the surface, thereby reducing the antisliding resistance of the soil. There are several types of rock slides, so I won’t introduce them one by one here. Generally speaking, rock sliding is plane sliding. It means that when the rock slides along the plane, the plane is more prone to plane sliding when the inclination angle of the sliding surface is greater than the internal friction angle. Two conditions need to be satisfied for the plane sliding of the slope rock mass, that is, to overcome the resistance on both sides and the resistance at the bottom. In soft rock, when the bottom inclination angle of the slope rock mass is much larger than the internal friction angle of the rock mass in the open-pit mine slope rock mass, the lateral restraint of the rock mass cannot provide enough force to prevent the rock from being damaged. Will detach from the slope rock mass to produce plane sliding. In the hard rock slope rock mass, only when the discontinuous surface of the slope rock mass crosses the top of the slope, and the rock on the slope is separated from the rock on both sides, the slope rock mass without lateral restraint may also slide in a plane^16^.

**Figure 3:**
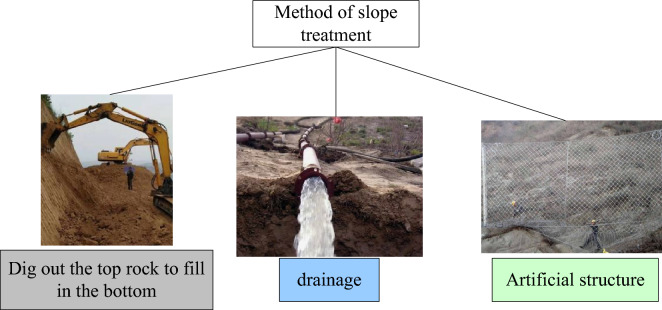
Methods of treating slopes.

Rock mass characteristics are another tool for classifying slopes, especially in mines. SMR is the most common classification scheme and is often used by different researchers to analyze the stability of cutting slopes in different mines. Slope quality rating is the main tool for understanding the rock mass behavior of open-pit mine slopes. Due to the increase in depth and slope angle, slope quality rating always brings serious problems. Due to various geological complexities, stability issues are more severe. The stability analysis of the moving slope was conducted using the Stereonet diagram. It is a simple tool to analyze wedge failure in planar and rock slopes. This structural data is geometrically plotted to establish the failure probability of the equal area network in the pattern^17^. There are also many ways to control the slope, there are generally three methods. As shown in Fig. 3.

From Fig. 3, it can be clearly seen that these three methods of slope management^18^. The first method is to dig up and make up. The general meaning is that there will be many rock masses with poor stability near the upper part of the slope. These rock masses with poor stability can be dug up, transported to the foot of the slope, and compacted. This can effectively enhance the stability. However, because the traction between the rock masses is still very strong, only the rock masses with poor stability can be dug up. The second method is drainage. Because rain is an essential reason for affecting stability. The accumulation of rainwater will affect the slippage of the cracks on the rock surface, resulting in the occurrence of landslides. Especially in the treatment of high, steep, and large slopes, drainage is particularly important. The third method is to use artificial structures for reinforcement. Anchor cables are generally used for protection and reinforcement. Of course, there are also retaining walls and antislide piles. All three methods work well. When controlling slopes, they can be used in combination to achieve better results^19^.

Moreover, slope material is important or slope geometry is important. Classified by stratigraphic lithology: it can be divided into soil slopes and rock slopes. (a) According to the rock structure, it is divided into layered structure slope, block structure slope, and network structure slope; (b) According to the relationship between rock strata inclination and slope direction, it can be divided into forward slope, reverse slope, and vertical slope. All slope instability involves the failure of slope rock and soil under shear stress. Therefore, the factors that affect the shear stress and the shear strength of rock and soil all affect the stability of the slope.

### Introduction to deep learning

**Figure 4:**
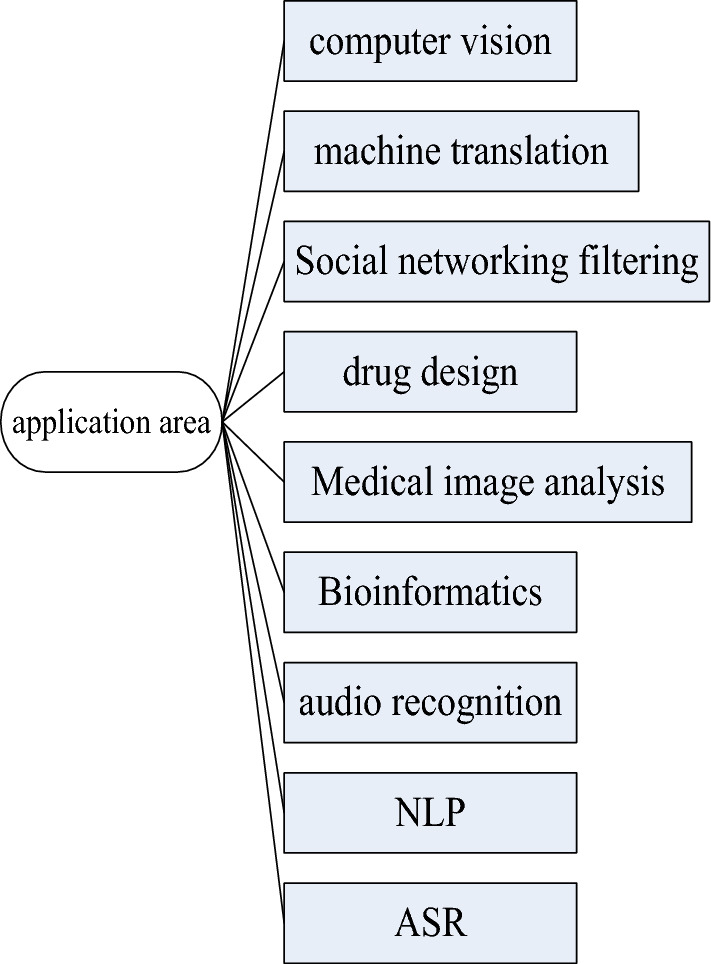
Application areas of deep learning.

Deep learning is actually a kind of machine learning method, and its predecessor is machine learning and artificial neural network^20^. However, because of the passage of time, this method is constantly developing and optimizing, and its application fields are also very wide. Specifically as shown in Fig. 4.

As shown in Fig. 4, this method has many application fields. The author lists nine areas in total. First of all, in the field of computer vision, this method can help computers process image data, or recognize text, and convert these images or text, which is very intelligent and convenient^21^. In the field of speech recognition, with the support of this algorithm, the efficiency of speech recognition has been greatly improved. Just like processing image data in computer vision, the method can turn sounds into recognizable models very quickly. In the field of audio recognition, this method is also used to improve the efficiency of audio recognition. In terms of social network filtering, the components in the network are very messy, and there are all kinds of information, but this method is very good at information classification, so this method is also very suitable for filtering social networks. In terms of machine translation, using this method can improve the quality of machine translation and make machine translation more inclined to the translation level of an ordinary person. In drug design, this method can assist the development of small molecule drugs, provide new computational decisions for pharmaceuticals, and process more chemical data information. In bioinformatics, using this method can bring new changes to the discipline. Because the method is so good at mining data, it is well suited for solving genomics problems. In the field of medical image analysis, after applying this method, a fast and very detailed analysis of medical images can be performed better. Because this method has already achieved good results in image segmentation. Therefore, it is also very suitable for image analysis in the field of medical images^22^.

**Figure 5:**
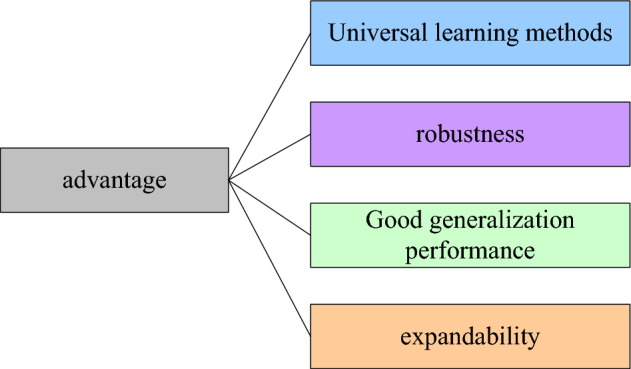
Advantages of deep learning.

The deep learning method not only has a wide range of applications, but also has many advantages, as shown in Fig. 5.

As shown in Fig. 5, its first advantage is high versatility. Generally, the data we deal with are multidimensional ordered data, and then due to the rapid development of big data, deep learning has been applied very well in various fields^23^. In addition to speech recognition and image classification, it also has very good performance in data mining and data processing and data prediction, so its versatility is high. The second advantage is robustness, which means that the method is smarter and more stable^24^. It can automatically adjust parameters according to data changes and automatically adapt to data changes. The third advantage is a good generalization. After the data is increased, it can still have good generalization ability, and the performance is not weakened at all, but enhanced. The fourth advantage is scalability, because when the neural network is stacked too much, the gradient will disappear or the gradient will explode, and this method can solve this problem very well. And this method has very good scalability in the number of layers and structural parameters and can be freely combined to achieve a better learning effect^25^.

**Figure 6:**
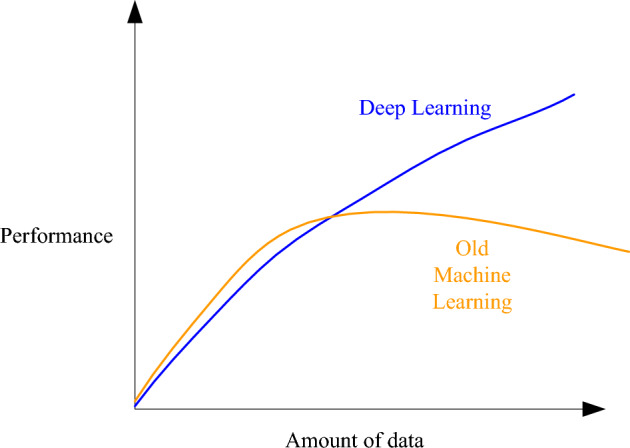
The relationship between the amount of training data and training performance.

In addition, the method can be generalized to the neural network structure trained in different fields, and can also have a good training effect in the case of insufficient data. We can compare the performance of machine learning and deep learning at training time, as shown in Fig. 6.

As shown in Fig. 6, it is obvious that the previous machine learning has too few parameters, and when the training data increases, the generalization ability will decrease^26^. The method proposed in this paper, when the training data increases, the generalization ability is better. This shows that the method proposed in this paper not only has good stability in data processing, but also can process very well data at the same time. Not affected by the size of the data volume.

Since deep learning is widely used, its framework has also been introduced by scholars. The code of the framework itself is very concise, the supported language types are quite rich, the technical documentation is complete, and the maintenance and operation are in good condition. Below I will list five more popular frameworks, as shown in Table 1.

**Table 1:** Frame display.

Serial number	Frame	Organization	Support language
1	Tensor Flow	Google	Python/C + + /Go/
2	Caffe	BVLC	C + + /Python
3	Torch	Facebook	Lua
4	CNTK	Microsoft	C +
5	Keras	fchollet	Python

As shown in Table 1, there are actually many mainstream frameworks, but for the convenience of analysis, the author lists the five most popular frameworks. The first framework is Tensor Flow, and its core code is written in C++. This is generally used to deal with multidimensional vectors. This framework also has visualization tools that can fully display the structure and data flow of the neural network. It contains many mainstream algorithms, and the entire design process is also comprehensive, which is very suitable for prediction in the industry. The second framework is Caffe, which defines each neural network. After ensuring normal docking, the network construction work is just stacking each layer. And it can participate in training as long as the model is defined, and the training performance is very good. The third framework is Torch, whose popularity is mainly due to the support of Facebook. This framework supports a lot of scientific computing, and it is generally the first choice for scientific research in academia. The fourth is CNTK, which is introduced by Microsoft. There are also many features, mainly the network structure is very fine, the code is product-level, and can be trained on a variety of hardware. The fifth framework is Keras. Its components are highly encapsulated. It is generally used by beginners, and it is relatively quick to get started. After understanding the principle, you can initially build a network.

### Deep learning algorithms (Dl)

(1) Recurrent neural network (rnn).

It is mainly a neural network generated to process sequence data.

Assuming the time is Y, you can get the model output as:

1 $${P}_{Y}=B\cdot {J}_{Y}+{N}_{P}$$

When the model predicts the output value at time Y, we analyze the loss function, and the backpropagation starts from the final loss value. Then during backpropagation, R represents the cost function, and the defined objective function is:

2 $$R=V\sum_{Y=1}^{Y}{\Vert {A}_{Y}-{U}_{Y}\Vert }^{2}=V{\sum }_{Y=1}^{Y}\sum_{K=1}^{A}{({A}_{Y}\left(K\right)-{U}_{Y}\left(K\right))}^{2}$$

The weight formula E can be updated by adjusting the cost function to be smaller:

3 $${E}^{NEW}=E-\rho \frac{\vartheta R}{\vartheta E}$$

ρ represents the learning efficiency, which can control the speed of parameter update. If it is not properly controlled, it will cause the optimization speed to not keep up. So to calculate the gradient, the error can be formulated as:

4 $${\varepsilon }_{Y}^{U}\left(K\right)=-\frac{\vartheta R}{\vartheta {B}_{Y}\left(K\right)}$$

5 $${\varepsilon }_{Y}^{J}\left(K\right)=-\frac{\vartheta R}{\vartheta {I}_{Y}\left(K\right)}$$

Then recursively calculate them, and the new formula can be obtained as:

6 $${U}_{Y}=H\left({E}_{JU}G\left({E}_{CJ}{C}_{Y+}{E}_{JJ}{J}_{Y-1}\right)\right)$$

7 $$R=V{\sum }_{Y=1}^{Y}{\Vert {A}_{Y}-{U}_{Y}\Vert }^{2}=V\sum_{Y=1}^{Y}{\left({A}_{Y}\left(K\right)-{U}_{Y}\left(K\right)\right)}^{2}$$

Y represents the last time point, at which the hidden layer can be expressed as:

8 $${\varepsilon }_{Y}^{J}\left(K\right)=-\left(\sum_{O=1}^{A}\frac{\vartheta R}{\vartheta {B}_{Y}(O)}\frac{\vartheta {B}_{T}(0)}{\vartheta {J}_{Y}(O)}\frac{\vartheta {J}_{Y}(K)}{\vartheta {I}_{Y}(K)}\right)$$

By derivation of this formula, the error formula at other time points can be obtained as:

9 $${\varepsilon }_{Y}^{J}\left(K\right)=\left({O}_{Y}\left(K\right)-{U}_{Y}\left(K\right){H}{\prime}\left({B}_{Y}\left(K\right)\right)\right)$$

The error formulas in the output layer and hidden layer are:

10 $${\varepsilon }_{Y}^{J}\left(K\right)=\left[\sum_{O=1}^{M}{\varepsilon }_{Y+1}^{J}\left(O\right){E}_{HH}\left(O,K\right)+\sum_{O=1}^{L}{\varepsilon }_{Y}^{U}\left(O\right){E}_{JU}\left(O,U\right)\right]{G}{\prime}\left({I}_{Y}\left(K\right)\right)$$

11 $${\varepsilon }_{Y}^{J}=\left[{E}_{JJ}^{Y}{\varepsilon }_{Y+1}^{J}+{E}_{JU}^{Y}{\varepsilon }_{Y}^{U}\right]\cdot {G}{\prime}\left({I}_{Y}\right)$$

It represents the error at time point Y and represents the error at time Y + 1.

This way, the weights of the output layer can be updated as:

12 $${\mathrm{E}}_{\mathrm{JU}}^{\mathrm{NEW}}\left(\mathrm{O},\mathrm{K}\right)={\mathrm{E}}_{\mathrm{JU}}\left(\mathrm{O},\mathrm{K}\right)-\upbeta {\sum }_{\mathrm{Y}=1}^{\mathrm{Y}}{\upvarepsilon }_{\mathrm{Y}}^{\mathrm{U}}\left(\mathrm{O}\right){\mathrm{J}}_{\mathrm{Y}}\left(\mathrm{K}\right)$$

The weights of the input layer can be updated as:

13 $${E}_{CU}^{NEW}\left(O,K\right)={E}_{CU}\left(O,K\right)-\beta {\sum }_{Y=1}^{Y}{\varepsilon }_{Y}^{J}\left(O\right){C}_{Y}\left(K\right)$$

The weights of the recurrent layer can be updated as:

14 $${E}_{JJ}^{NEW}\left(O,K\right)={E}_{JJ}\left(O,K\right)-\beta {\sum }_{Y=1}^{Y}{\varepsilon }_{Y}^{J}\left(O\right){J}_{Y-1}\left(K\right)$$

(2) Long short-term memory neural network (lstm)

Although it is similar in structure to RNN, it will change the increased cell state in the structure according to the existence time, and this cell state is a long-term memory^27^. The LSTM algorithm is often used to perform operations such as prediction of various data or image recognition. Its forgetting gate determines whether the knowledge I have already learned is useful, and which part I want to discard; the input gate determines whether the knowledge others tell me is useful to me, and which knowledge I want to receive; integrating my current knowledge through the output gate some knowledge determines what to report to others^28^. The theory and learning process of LSYM are valuable, and it has good problem-solving ability when solving some practical problems.

If it is a parameter in the output gate, it is the output of the hidden layer. The formula of the input gate can be obtained as:

15 $${O}_{Y}=\tau \left({E}_{CO}{C}_{Y}+{E}_{DO}{D}_{Y-1}+{E}_{VO}{V}_{Y-1}+{N}_{P}\right)$$

16 $${D}_{Y}={P}_{Y}TANH({V}_{Y})$$

At this time, if you want to update the cell state, the formula can be expressed as:

17 $${V}_{Y}={G}_{Y}{V}_{Y-1}+{O}_{Y}{V}_{Y}$$

18 $$\overline{{V }_{Y}}=\tau \left({E}_{O}\cdot \left[{J}_{Y-1},{C}_{1}\right]+{N}_{O}\right)$$

Calculated according to the state of the current time, the final output value can be obtained.

19 $${P}_{Y}=\tau \left({E}_{CP}{C}_{Y}+{E}_{DP}{J}_{D-1}+{N}_{P}\right)$$

20 $${D}_{Y}={P}_{Y}TANH({V}_{Y})$$

## Experiment and analysis on prediction of slope stability coefficient of open pit mine based on deep learning

### Algorithm prediction performance comparison

According to the information obtained, many algorithms have been used to predict slope stability. So we compared them with our algorithm on the test set for a prediction performance comparison. The results are shown in Table 2.

**Table 2:** Algorithm performance comparison.

By the end of the baseline					Youden baseline		
		Stability	Instability	Accuracy	Stable value	Unstable value	Accuracy
LR	Stability	18	4	0.80	18	4	0.82
	Instability	7	20		6	19	
DT	Stability	17	5	0.79	16	6	0.90
	Instability	7	20		5	20	
DL	Stability	20	0	0.94	20	0	0.94
	Instability	4	21		4	21	

As shown in Table 2, the accuracy and stability of each algorithm calculation when using the baseline and Youden cutoff points. It can be seen that the performance of the Youden cutoff is generally better than that of the baseline cutoff. The average accuracy of all algorithms increases by 4.8% from the baseline cut-off point to the Youden cut-off point. The largest increase in accuracy is achieved through the DT algorithm, and after using Youden, the accuracy increases from 79 to 90%. There is a 0.2% increase in accuracy in the best LR algorithm at the Youden cutoff, while the best DL algorithm has no accuracy change. The highest accuracy was achieved by the optimal DT algorithm with the Youden cutoff, in this case 43 out of 45 slope cases were correctly predicted. The accuracy of the best DT algorithm and the best DL algorithm for the Youden cutoff is greater than 90%.

**Figure 7:**
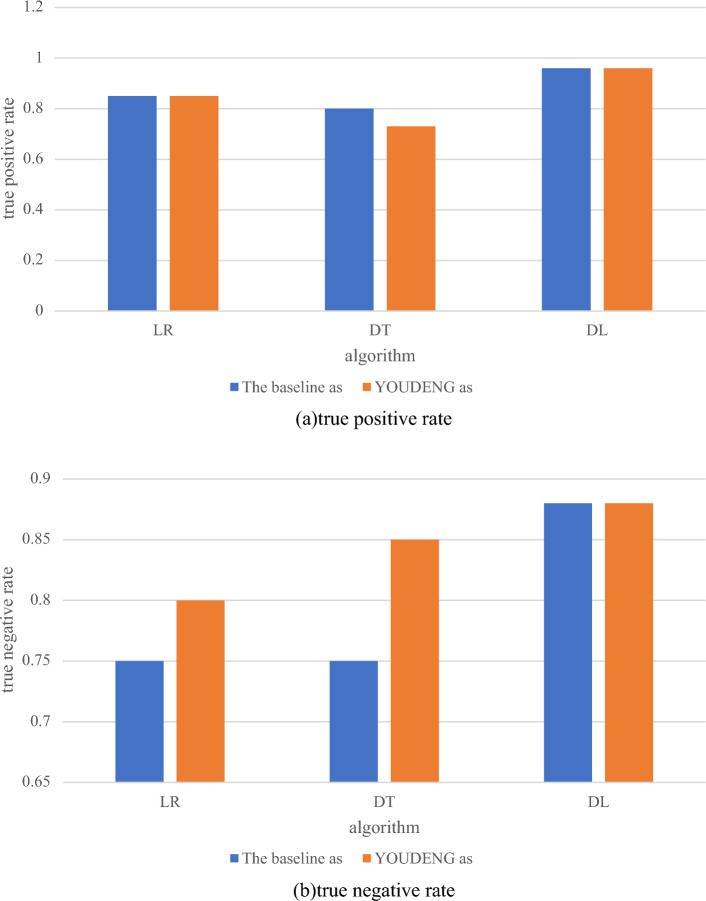
Test performance.

Finally, their predictive performance is compared by the true positive rate and the true negative rate they show on the test set. The results are shown in Fig. 7.

As can be seen from Fig. 7, the best classification algorithm for correctly predicting stable slopes is the DL algorithm. Under both algorithms, all 20 stable slopes are correctly measured and compared to the optimal DT that passes the cutoff point. The algorithm achieves the optimal prediction of slope stability and correctly predicts all unstable slope cases. Baseline and Youden's cutoff have no effect on the performance of the optimal DL model in this paper^29^.

In order to better verify the rationality and reliability of the DL algorithm used in this paper, the author uses three methods to calculate the safety factor for the slope examples in many literatures. The calculation results are shown in Table 3.

**Table 3:** Safety factor calculation results.

Calculation method	Traditional method		DL
	Peak strength	Residual strength	
Example 1	1.315	1.121	1.236
Example 2	1.030	0.534	0.958
Example 3	1.404	1.083	1.2973

It can be seen from Table 3 that the safety factor value of the DL algorithm is between the peak strength and residual strength of the traditional algorithm. In Example 1, the safety factor values of the traditional algorithm for the two strengths are 1.315 and 1.121, respectively. The safety factor of the algorithm in this paper is 1.236. In Example 2, the safety factor values of the traditional algorithm are 1.03 and 0.534, respectively. The safety factor of the algorithm in this paper is 0.958. In Example 3, the safety factor values of the traditional algorithm are 1.404 and 1.083, and the safety factor value of the algorithm in this paper is 1.2973. The research shows that the safety factor obtained by the traditional algorithm is actually very dangerous in practical application by using the peak strength, and the safety factor obtained by the traditional algorithm by using the residual strength is relatively conservative^30^.

### Stability coefficient prediction experiment

The National Highway (NH-305) is crucial as it is used as the only alternative connection for the transportation of military goods and other materials during the closure of other highways. Many slope failures have been reported in the past, so hazard zoning maps were developed using five commonly used parameters to identify potential vulnerable areas. Subsequently, a detailed on-site investigation was conducted to collect rock engineering parameters and geomechanical classification. Several locations were identified from the hazard zoning map and subsequent on-site investigations for stability analysis. The classification of the danger zone clearly classifies the steep slope on the right bank of the Sutlej River as a potential damage zone, which is also confirmed by the low quality rating of the slope. The finite element method was later used to study the deformation mechanisms related to the failure of such slopes. The high value of the safety factor indicates the stability of the slope, but the displacement contour and shear strain concentration near the foot of the slope indicate that this is not the case^31^. We take an open-pit mine as an example and use the algorithm in this paper to predict, calculate, and analyze the stability coefficient of the slope. First, make sure that the slope is in a stable state after treatment, and then analyze the stability of the slope under heavy rain^32^.

**Figure 8:**
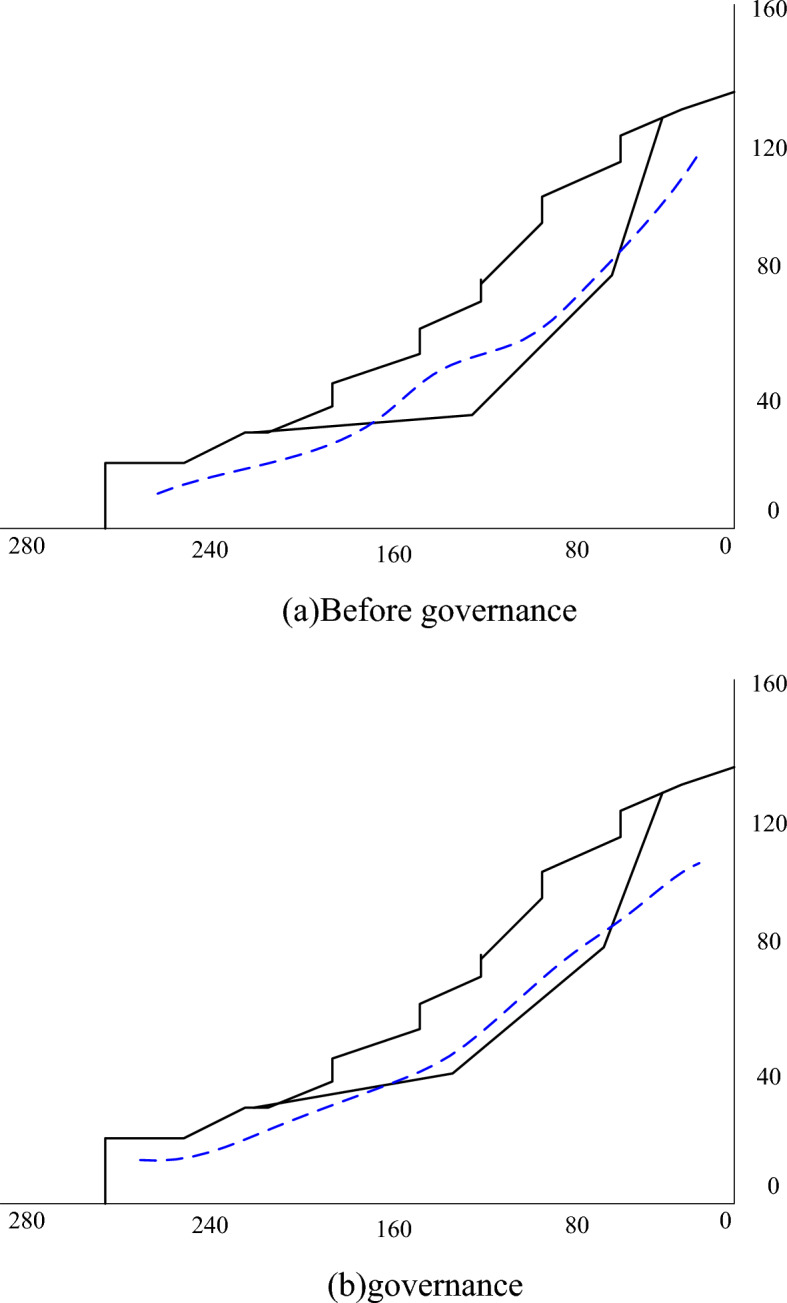
Sectional view before and after landslide treatment.

According to the shape of the landslide and the main sliding direction of the landslide, the 129 line of the main sliding section of the landslide is selected as the calculation section, and the sliding surface exposed by drilling is used as the calculation sliding surface. The water in the site is mainly of shallow upper layer stagnant water and deep bedrock fissure water. According to the measurement of each borehole in the Jiepailing Mine site survey, the mixed stable groundwater level is located at a buried depth of about 24 m. The calculated section of the landslide before treatment and the calculated section after the landslide has been reduced to + 521 m are shown in Fig. 8.

**Figure 9:**
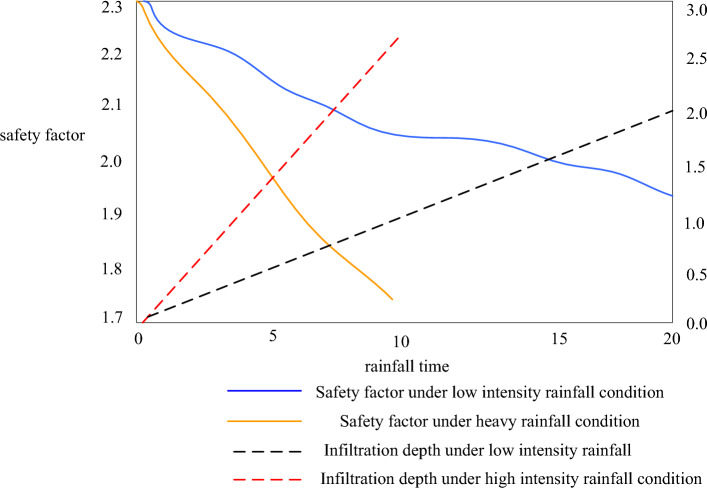
Prediction of slope safety factor under rainfall.

We studied the variation of the slope safety factor and infiltration depth with rainfall duration using the DL algorithm under two rainfall conditions. The obtained results are shown in Fig. 9.

In the case of considering the matrix suction, the initial slope safety factor before rainfall increased from 1.25 to 2.4. It can be seen that the matrix suction can make a great contribution to the slope stability, and the slope stability calculation without considering the matrix suction is conservative. Under the two rainfall conditions, the safety factor decreases rapidly with the rainfall time. It can be seen that rainfall has an obvious impact on the slope stability, and long-term rainfall is likely to induce landslides. This also reflects that the DL algorithm is very suitable for calculating the prediction of the slope stability coefficient.

**Figure 10:**
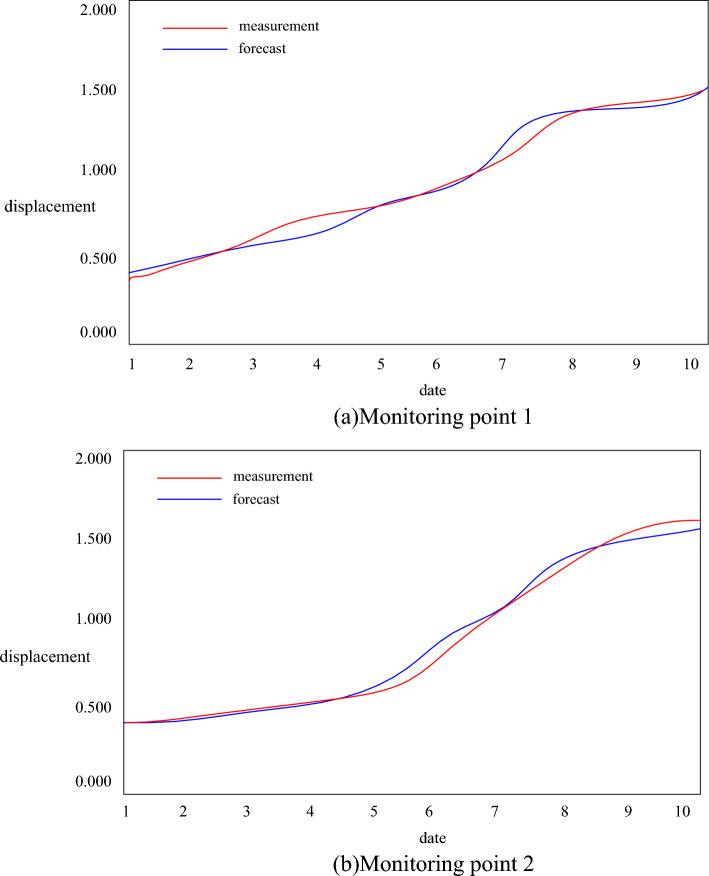
Measured and predicted values of monitoring points.

In order to verify the reliability of the analysis method, the validity of the existing project monitoring data prediction method is adopted. Take the data of a project as the data source. Using the IoT system monitoring data and data analysis experimental platform based on the deep learning algorithm to study the variation law of the displacement of high and steep slopes. Considering the influence of the slope rock mass by underground and ore body mining, a total of 2 monitoring points were set up to monitor it in real time using a total station. The DL model is used to model and analyze the real-time measured displacement of the slope monitoring point, and the results are shown in Fig. 10.

The structural deformation of mining engineering involving high and steep slopes has the characteristics of ambiguity and nonlinearity. It is difficult to accurately express and predict the evolution trend of deformation and the occurrence area and time of the instability state through numerical analysis and similar model experiments. It will be comprehensively reflected in the deformation of the monitoring point. The DL algorithm can solve the problems of small samples, nonlinearity and high dimensionality very well, and the formed DL model has high prediction accuracy. As can be seen from Fig. 10, the DL model can accurately fit the sample data set and has the prediction ability of the same time series as the sample data. Using ten days of measured data to build learning samples for modeling to predict the stability of monitoring points, and found that the maximum error is 3.12%. It can be seen that the stability prediction based on the DL algorithm can fully meet the engineering requirements for accuracy, improve the efficiency of mine development, and provide a safety reference for mine development.

Next, we will use the DL algorithm to analyze the stability calculation results of a mine. The safety factor of the slope is calculated using the traditional algorithm and the algorithm of this paper, respectively. The results are shown in Table 4.

**Table 4:** Safety factor prediction calculation results.

Working condition of calculation	Safety factor	
	Traditional algorithm	DL
Pre-treatment profile	1.167	1.086
Cut slope + 521 profile	1.303	1.227

It can be seen from Table 4 that the safety factor calculated by the algorithm in this paper is about 7% lower than that of the traditional algorithm. In the stability analysis of the slope before treatment, the safety factors calculated by the traditional algorithm and the algorithm in this paper are 1.167 and 1.086, respectively. The former is higher than the safety factor requirement of 1.15 specified in the specification, but the actual slope is damaged, which proves that the the limit equilibrium method of progressive destruction is relatively safe, and the algorithm in this paper is closer to reality. In the stability analysis of the slope after treatment, the safety coefficients calculated by the traditional algorithm and the algorithm in this paper are 1.303 and 1.227, respectively, and the stability coefficients meet the requirements of the specification, indicating that the treatment plan for slope reduction and load reduction has achieved remarkable results.

In addition to the comparison between the traditional algorithm and the algorithm in this paper. We also predicted the stability system of the landslide using the DL algorithm under rainfall conditions. The results are shown in Table 5.

**Table 5:** Stability analysis under different rainfall scenarios.

Scheme	Rainfall intensity(m/min)	Duration of rainfall(h)	Safety factor
Plan a	$1.95\times {10}^{-5}$	24	1.0929
Plan b	$8.29\times {10}^{-5}$	24	1
Plan c	$2.06\times {10}^{-5}$	24	1

It can be seen from Table 5 that the safety factor of the slope is lower than 1.15 after the rainfall intensity of Schemes 1, 2, and 3 lasts for 24 h, and even lower than 1 in Schemes 2 and 3, the slope is about to fail, and the corresponding measures need to be taken measure. This also shows that the application of DL algorithm is very suitable for the prediction of slope stability coefficient. And the predicted value is in line with the actual situation.

**Figure 11:**
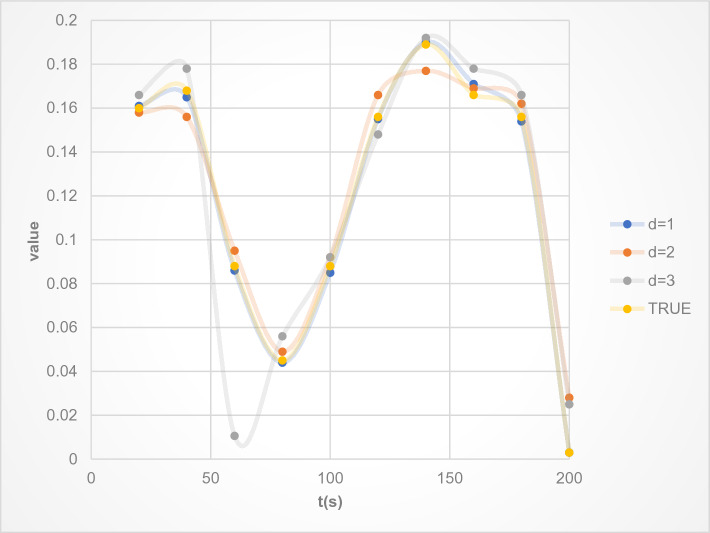
The predicted slope angle results of deep learning in this paper.

In order to compare the error calculations, this article conducts experimental comparisons between the existing LSTM model and STL-LSTM, as well as the proposed deep learning model. This article constructs datasets for d = 1, 2, and 3, respectively, the predicted slope angle results are shown in Fig. 11, and the error calculation results are shown in Table 6.

**Table 6:** Error calculation results.

Model types	d = 1	d = 2	d = 3
LSTM	0.0089	0.0157	0.0263
STL-LSTM	0.0056	0.0112	0.0189
Deep learning in the paper	0.0032	0.0096	0.0123

From Fig. 11, it can be seen that the predicted value of the slope angle is basically not significantly different from the actual value, and when d = 1, the predicted value is basically equal to the actual value, which can prove the usability of the deep learning model developed in this paper.

## Conclusions

In this paper, the deep learning algorithm is used to study and analyze the trend of slope stability coefficient prediction in open pit mines. Prediction of stability is an essential link in mine development, which will affect the production efficiency and safety factor of the entire mine. Therefore, you should try to use deep learning algorithms for calculations when making stability predictions, so that the obtained results are closer to reality. It can be seen that the performance of the Youden cutoff is generally better than that of the baseline cutoff. Due to the limited space of the article, it cannot cover all aspects. At the same time, there are not many examples used in the study. This algorithm is based on artificial intelligence deep learning algorithms and is built on certain assumptions. If these assumptions are not true, it will affect the predictive ability of the model. This is also the limitation of this article. In the future, the author looks forward to using more real data to conduct deeper research to excavate more and predict slopes.

## Data Availability

The datasets used and/or analysed during the current study are available from the corresponding author on reasonable request.
